# In Situ Collection and Preservation of Intact *Microcystis* Colonies to Assess Population Diversity and Microcystin Quotas

**DOI:** 10.3390/toxins11080435

**Published:** 2019-07-24

**Authors:** Jonathan Puddick, Eric O. Goodwin, Ian Hawes, David P. Hamilton, Susanna A. Wood

**Affiliations:** 1 Cawthron Institute, Nelson 7010, New Zealand; 2 University of Waikato Coastal Marine Field Station, Sulphur Point, Tauranga 3110, New Zealand; 3 Australian Rivers Institute, Griffith University, Brisbane 4111, Australia

**Keywords:** cryo-sampling, cyanobacteria, cyanotoxin, high-throughput sequencing, internal transcribed spacer region, liquid chromatography-mass spectrometry, quantitative polymerase chain reaction

## Abstract

Understanding of colony specific properties of cyanobacteria in the natural environment has been challenging because sampling methods disaggregate colonies and there are often delays before they can be isolated and preserved. *Microcystis* is a ubiquitous cyanobacteria that forms large colonies in situ and often produces microcystins, a potent hepatotoxin. In the present study a new cryo-sampling technique was used to collect intact *Microcystis* colonies in situ by embedding them in a sheet of ice. Thirty-two of these *Microcystis* colonies were investigated with image analysis, liquid chromatography-mass spectrometry, quantitative polymerase chain reaction and high-throughput sequencing to assess their volume, microcystin quota and internal transcribed spacer (ITS) genotype diversity. Microcystin quotas were positively correlated to colony volume (*R*^2^ = 0.32; *p* = 0.004). Individual colonies had low *Microcystis* ITS genotype diversity and one ITS operational taxonomic unit predominated in all samples. This study demonstrates the utility of the cryo-sampling method to enhance the understanding of colony-specific properties of cyanobacteria with higher precision than previously possible.

## 1. Introduction

*Microcystis* is a common genus of bloom-forming freshwater cyanobacteria that is frequently associated with the production of the cyanotoxin microcystin [1]. It is characterised by small cells (ca. 1–8 µm) that often aggregate into large colonies [2]. Colonies, potentially made up of hundreds of thousands of cells, are held together in an organic matrix comprised of a range of secreted organic polymers such as polysaccharides, nucleic acids, phospholipids and/or proteins [3,4]. Microcystins are cyclic heptapeptides, which are non-ribosomally synthesised by a multifunctional enzyme complex [5] and more than 250 microcystin structural congeners have been described to-date [6]. The toxins predominantly affect the liver cells of mammals as they cannot be translocated across the membranes of most tissues and are actively transported into hepatocytes [7] by organic anion-transporting polypeptides [8]. Inhibition of hepatocyte protein phosphatases results in excessive signaling, leading to cellular disruption due to intermediate filaments of cytokeratin in the cytoskeleton becoming hyperphosphorylated [9], or cell proliferation and tumor promotion [10].

Previous research has identified a positive relationship between *Microcystis* colony size and microcystin production. Kurmayer et al. [11] used a sieving procedure to separate natural populations of *Microcystis* colonies into different size classes. They showed that larger colony size classes contained a greater proportion of cells with microcystin-producing genotypes and had higher microcystin quotas (i.e., the amount of microcystin per cell). These observations corroborate the results of an experiment conducted by Gan et al. [12], where *Microcystis* cultures supplemented with extracellular microcystin developed larger colonies than control cultures. Colony formation benefits cyanobacteria through increased buoyancy as cell diameter increases [13], which in turn enables colonies to access higher light levels at the water surface [14,15] and to reduce predation [16,17].

A cryo-sampling technique was recently developed to collect and preserve cyanobacteria samples in situ [18]. Metal probes chilled with liquid nitrogen were used to almost instantaneously freeze samples. The cryo-sampling overcomes conventional sample processing artefacts of colony disaggregation through sieving and delay between sample collection, colony isolation and preservation. The cryo-sampling technique also allows for the accurate determination of colony size and for individual colonies to be assessed. In the present study, *Microcystis* colonies were collected in situ using a ‘surface snatcher’ cryo-sampler to test the hypothesis that larger cyanobacteria colonies have higher microcystin quotas and to evaluate the internal transcribed spacer (ITS) genotype composition of individual *Microcystis* colonies.

## 2. Results

*Microcystis* colonies were collected in situ from three locations at Lake Rotorua (Kaikoura, New Zealand) using a cryo-sampling technique designed to encapsulate cyanobacterial colonies in a sheet of ice. Thirty ice sheets were collected and preserved frozen. The volume of 32 frozen *Microcystis* colonies was determined by image analysis (Supplementary Table S1) before colonies were individually removed from the ice sheets. Subsets of the samples were assessed for microcystin quota (*n* = 12), the diversity of the *Microcystis* population (*n* = 8) or both (*n* = 12).

**Figure 1:**
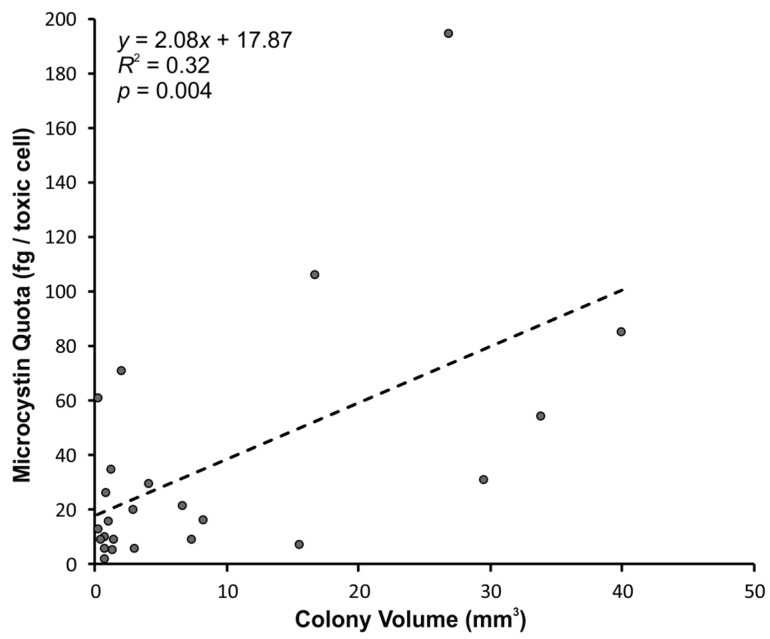
The relationship between microcystin cell quota and *Microcystis* colony size for samples collected in situ using the surface snatcher cryo-sampler.

Microcystins were detected in all of the colony samples analysed using liquid chromatography-tandem mass spectrometry (LC-MS/MS; Supplementary Table S2). Two microcystin congeners were present; dmMC-LR in higher concentrations and didmMC-LR in lower relative concentrations. Microcystin quotas of the cells in the colonies were positively correlated to colony volumes (*p* = 0.004, *R*^2^ = 0.32; Figure 1). A relationship was also observed between the log_10_-transformed data (*p* = 0.02, *R*^2^ = 0.22). Microcystin quotas of colonies from different collection sites/dates were not significantly different (*p* = 0.17).

**Figure 2:**
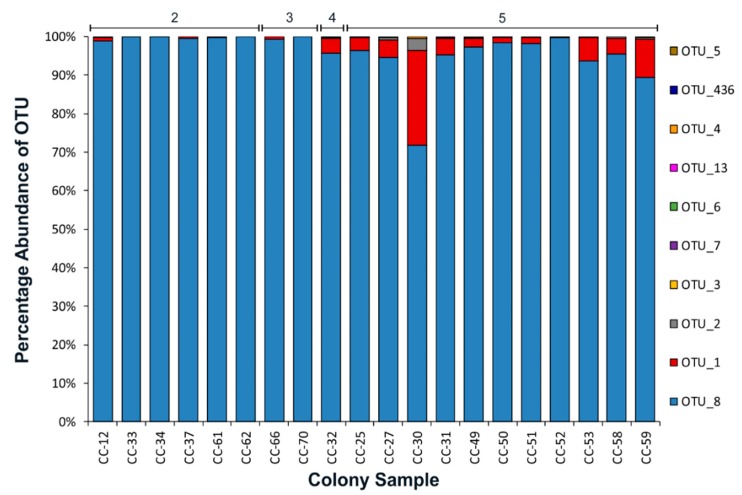
Relative abundance of internal transcribed spacer gene operational taxonomic units (OTUs) of 20 *Microcystis* colonies collected in situ using the surface snatcher cryo-sampler (site numbers are provided at the top of the chart).

The *Microcystis* ITS genotype composition of 20 colonies was assessed by high-throughput sequencing (HTS). Ten operational taxonomic units (OTUs) from the genus *Microcystis* were observed in the samples, although seven of the OTUs each comprised ≤ 0.5% of the sequence reads for an individual colony (OTU_3, OTU_4, OTU_5, OTU_6, OTU_7, OTU_13 and OTU_436; Figure 2). Three OTUs (OTU_1, OTU_2 and OTU_8) comprised ≥ 1% of the sequence reads of an individual colony. Of these, OTU_8 comprised ≥ 71% of the sequence reads in the colonies (assessed by HTS) and > 99% of the sequence reads in nine of the colonies (CC-33, -34, -37, -52, -61, -62, -63, -66, -70).

There was no relationship between colony size and proportion of reads for OTU_1 (*p* = 0.31) or OTU_2 (*p* = 0.46). The *Microcystis* ITS sequence diversity in the individual colonies was affected to some degree by collection site/time. For example, OTU_8 comprised 98.9% of the reads in colonies collected from Collection 2 (Boat Bay) and ≥ 99.4% in the colonies collected from Collection 3 (Pontoon; Supplementary Table S3). These samples were collected from the South-eastern side of Lake Rotorua on 13 April 2014. Collections on the South-western side of the lake were conducted on 14 April 2014 (Launch Bay) and contained greater *Microcystis* population diversity with lower proportions of OTU_8 and higher proportions of OTU_1 (0.1–24.7%) and OTU_2 (0.1–3.1%) sequence reads. When only samples from Site 5 (where the greatest level of ITS genotype diversity was observed) were assessed, there was still no relationship between colony size and proportion of reads for OTU_1 (*p* = 0.25) or OTU_2 (*p* = 0.47).

## 3. Discussion

Using cryo-samplers, we investigated the hypothesis that larger *Microcystis* colonies have higher microcystin quotas. Previous research by Kurmayer, Christiansen and Chorus [11] showed that the *Microcystis* cells of larger colony size classes from Lake Wannsee (Germany) had higher microcystin quotas. The results achieved using the cryo-samplers [18] confirmed this previous observation, but the ability to determine the colony volume (rather than a size class) allowed the relationship between colony volume and microcystin quota to be assessed more precisely. Whilst the relationship between colony volume and microcystin quota was statistically significant (*p* = 0.004), the relationship was not particularly strong (*R*^2^ = 0.32). This may be due to other factors, not assessed during the present study, influencing microcystin quotas. Cryo-sampling also minimised sampling artefacts associated with delay before sample preservation and colony disaggregation from sieving. Measuring the microcystin quota of individual colonies, instead of a composite sample from a certain size class, allowed assessment of the natural variability in microcystin quotas between colonies. One disadvantage of the cryo-sampling technique, however, is that only colonies on the water surface can be collected, therefore, our dataset is not representative of a vertically integrated sample but a surface sample instead.

HTS was used to explore the *Microcystis* ITS genotype composition of a subset of individual *Microcystis* colonies. Sequencing of the cyanobacterial ITS sequence has previously been used to evaluate changes in populations of cyanobacteria of the same species [19,20], as the gene is subject to high levels of sequence variation. In the present study *Microcystis* colonies collected from Lake Rotorua were generally dominated by a single ITS sequence. Similar findings were reported by Janse et al. [21] who used denaturing gradient gel electrophoresis (DGGE) of the cyanobacterial ITS sequence to assess *Microcystis* population diversity in colonies collected from Lake ‘t Joppe and Lake Zeegerplas (The Netherlands). Only one genotype of *Microcystis* was observed (a single DGGE band) in 72% of the colonies assessed by Janse et al. [21]. Multiple DGGE bands were observed in 28% of the colonies, which the researchers surmised might be due to aggregation of different *Microcystis* colonies or the presence of multiple rRNA operons containing different ITS sequences. The greater degree of diversity observed in our study was likely due to the increased sensitivity of HTS compared to DGGE and the use of cryo-samplers to collect the colony samples, as loosely bound colonies were not disaggregated during collection. In contrast to the work of Janse et al. [21], all of the colonies assessed in our study produced microcystins, although sample collection spanned several days compared with several months in the former study, and changes in *Microcystis* strain dominance were likely to increase with sampling duration.

The ITS genotype composition in the *Microcystis* colonies assessed from Lake Rotorua was not related to colony size but may have been affected by collection site or time. Colonies collected from the South-eastern side of Lake Rotorua on 13 April 2014 had lower ITS genotype diversity than colonies collected from the South-western side of the lake on 14 April 2014. Different populations of *Microcystis* may have been present in different parts of the lake providing different growing conditions. For example, the South-western side of the lake is a sheltered bay whereas the North-eastern side is not sheltered. The increased ITS sequence diversity observed in samples collected on 14 April 2014 may also have been due to conditions promoting or allowing different *Microcystis* genotypes to sit at the water’s surface (compared to 13 April 2014), but further discrimination is not possible at this stage.

Briand et al. [20] successfully used ITS genotype compositions, obtained via sequencing of clone libraries, to assess shifts in *Microcystis* strain dominance at the Grangent Reservoir (France). They found that a single ITS genotype progressively became dominant, coinciding with a reduction in the abundance of microcystin-producing strains. Similar studies assessing individual colonies, using the cryo-samplers, and the broader *Microcystis* community, using water samples, would be valuable for better understanding *Microcystis* colony formation in the natural environment. 

Using cryo-samplers to collect and preserve *Microcystis* colonies in situ allowed more precise assessment of the relationship of microcystin quota to colony size and corroborated the results of previous studies. The ITS composition of the *Microcystis* colonies from Lake Rotorua was dominated by one ITS genotype. Future studies over longer time periods will provide more insight into the links between microcystin production and cyanobacterial colony formation. 

## 4. Materials and Methods 

### 4.1. Sample Collection

Samples were collected at Lake Rotorua (Kaikoura), a small (0.55 km^2^), shallow (max. depth 3 m), eutrophic lake in the northeast of the South Island of New Zealand (42°24’05S, 173°34’57E) [22]. Buoyant *Microcystis* colonies on the lake surface were collected using the ‘surface snatcher’ cryo-sampler (5 × 5 cm^2^) described in Puddick, Wood, Hawes and Hamilton [18]. The sampling device was cooled in liquid nitrogen until bubbling ceased and then held to the water surface adjacent to a *Microcystis* colony. Over 20 to 30 s an ice sheet formed progressively downwards from the water surface to a depth of ca. 3.5 mm in calm conditions. Ice sheets were removed from the sampling device with tweezers, laid on transparent plastic sheets, placed in a zip-lock bag and immediately stored on dry ice. Upon returning to the laboratory, the ice sheets were transferred to a −20 °C freezer until colony removal.

Five collections (Ice Sheets 1–5) were conducted between 12–14 April 2014 from three locations at Lake Rotorua (Supplementary Table S4). Multiple ice sheets were collected at each sampling point and were labelled in alphabetical order.

### 4.2. Image Acquisition, Colony Removal and Sample Extraction

Colony removal and image acquisition were conducted in a −20 °C walk-in freezer over a two-day period. Ice sheets were placed on a height-adjustable stand and images of single colonies were captured using a portable digital USB microscope (CollingTech; Guangdong, China). Following adjustment of the camera’s optical zoom, the image was focused by adjusting the distance between the ice sheet and the camera. Each colony image was followed by an image of a mm scale without altering the camera’s optical zoom settings or the height of the adjustable stand. Images were captured in JPEG format at a resolution of 480 × 360 pixels.

After image acquisition, the ice surrounding the colony was melted by placing an electrical soldering iron near the ice. The resulting water and cyanobacteria cells were then removed using a 200 µL autopipette and placed in a 200 µL tube. Melting the ice and removing the cells was repeated until the entire colony was removed. Pipette tips blocked when the water re-froze whilst working in the −20 °C freezer, so tips used for each sample were retained and the sample reclaimed by thawing at ambient temperature and briefly centrifuging in a 15 mL Falcon tube, to draw the liquid down into the collection tube.

The excised colonies were extracted for microcystins and DNA as described in Puddick, Wood, Hawes and Hamilton [18]. The samples were heated at 99 °C for 1 min in a polymerase chain reaction (PCR) thermal cycler (Eppendorf Mastercycler; Hamburg, Germany). An aliquot for microcystin analysis was placed in a 1.8 mL tube and an aliquot for molecular analysis was placed in a 200 µL tube. The aliquot for microcystin analysis was further extracted by sonication in 50% methanol + 0.1% formic acid (*v*/*v*) and the aliquot for molecular analysis was supplemented with Tween-20 before being re-heated to 99 °C for 1 min in the PCR thermal cycler.

### 4.3. Colony Image Analysis and Volume Estimation

Image analysis of colony images was conducted in R [23] using the EBImage package [24] and BioConductor [25]. Images were processed independently. The pixel area of each colony was determined first, then the pixel to mm conversion determined by calibration from the image of the mm scale. The colony area (in pixels) was determined by isolating the pixels that were darker in the blue and red channels than their respective Otsu thresholds [26] (as determined for the current image only), as well as having a higher green intensity than red intensity. The mask defined by these RGB intensity requirements was filtered by a 2-pixel median filter [27], followed by an erosion and dilation [28], each using a 5 × 5 diamond kernel. Colony images isolated by this selection technique were visualised alongside the original image to confirm and validate the selection rules (Supplementary Figure S1). The area of the colony was then determined as the area in pixels of the selection mask.

The volume of the colony was estimated by assuming the darkest point of each colony spanned the full thickness of the ice sheet, and that the relative thickness of the colony at other pixel locations was linearly related to the relative darkness of that pixel. All three colour channels contributed to this darkness value. The pixel to mm conversion for each image was then applied to convert the colony volume into mm^3^.

### 4.4. Quantitative Polymerase Chain Reaction Analysis

Enumeration of microcystin-producing *Microcystis* was conducted using a quantitative PCR (qPCR) assay targeting the microcystin synthase E (*mcyE*) gene involved in microcystin production. The procedures used are described in Puddick, Wood, Hawes and Hamilton [18].

### 4.5. Microcystin Analysis

Microcystin concentrations in excised colony samples were determined by LC-MS/MS as described in Puddick, Wood, Hawes and Hamilton [18]. When sample concentrations were outside of the standard curve, the samples were diluted with 50% methanol and re-analysed. Microcystin quotas (the amount of microcystin per toxic cell) were calculated by summing the concentration of all congeners observed in the samples and dividing by the level of toxic cells (determined using *mcyE* qPCR).

### 4.6. High-Throughput Sequencing

Twenty colonies were selected for analysis of the ITS sequence between the 16S and 23S rRNA genes (rRNA-ITS) using HTS. A region of ca. 500 bp was amplified using cyanobacterial-specific primers ULR [29,30] and CSIF [21], modified to include Ilumina™ adapters. PCR reactions were performed in 50 µL volumes containing 25 μL of AmpliTaq Gold^®^ 360 Master Mix (Life Technologies, Carlsbad, CA, USA), 5 μL CG inhibitor (Life Technologies), 0.5 μM of each primer, and template DNA (ca. 20 ng). PCR cycling conditions were: 95 °C for 10 min, followed by 27 cycles of 95 °C for 30 s, 50 °C for 45 s, 72 °C for 45 s, and a final extension of 72 °C for 7 min. PCR products were visualized with 1% agarose gel electrophoresis with RedSafe DNA Loading Dye (iNtRON Biotechnology, Seongnam, South Korea) and UV illumination. PCR products were purified (Agencourt^®^ AMPure^®^ XP Kit, Beckman Coulter, Brea, CA, USA), quantified (Qubit^®^ 20 Fluorometer, Invitrogen, Carlsbad, CA, USA), diluted to 10 ng/µL and submitted to the University of Auckland (New Zealand) for library preparation. Sequencing adapters and sample-specific indices were added to each amplicon via a second round of PCR using the Nextera™ Index kit (Illumina, San Diego, CA, USA). Libraries were sequenced on a MiSeq Illumina™ platform (2 × 300 reads). 

Overlapped raw sequence reads were de-noised, trimmed and filtered prior to downstream analyses. Paired-end reads were assembled into contigs using USEARCH [31]. Merged reads < 200 bp were discarded. The data were then filtered with VSEARCH [32], and reads with more than one expected error [33] per read were discarded. The data were then dereplicated with all non-unique sequences removed to make downstream computation faster. OTUs were generated using VSEARCH by clustering each unique sequence at the 99% identity threshold. Non-unique reads were then mapped back onto these clusters, and any cluster that contained fewer than 10 sequences was discarded. Taxonomy was then assigned to each OTU using a reference database, which was constructed using all available cyanobacteria ITS sequences from GenBank [34]. Only ITS sequences assigned to *Microcystis* were utilised for further analysis.

### 4.7. Statistical Analysis

One-way ANOVA tests and linear regression were conducted using the R stats package [23,35]. For one-way ANOVA tests, microcystin quota data and ITS genotype proportions were log_10_ transformed to fulfil the assumptions of normality and heterogeneity of variance.

## Supplementary Materials

The following are available online at https://www.mdpi.com/2072-6651/11/8/435/s1, Table S1: Image analysis information to determine colony volume, Table S2: Microcystin quota information for colony samples, Table S3: Sample information for high-throughput sequencing of the cyanobacterial internal transcribed spacer gene for relevant colony samples, Table S4: Information on sample collection time and location, Figure S1: Automated pixel selection for colony images where images alternate between the raw image and the selected area.
