# Supplementary material for: In Situ Collection and Preservation of Intact Microcystis Colonies to Assess Population Diversity and Microcystin Quotas
Source: Toxins (Basel). 2019 Jul 24;11(8):435. doi: 10.3390/toxins11080435 (PMC6722930; doi:10.3390/toxins11080435)

Supplementary Materials: In Situ Collection and Preservation of Intact *Microcystis* Colonies to Assess Population Diversity and Microcystin Quotas

Jonathan Puddick*, Eric O Goodwin, Ian Hawes, David P Hamilton and Susanna A Wood

Table 1. Image analysis information to determine colony volume.

Image ID	Colony ID	Ice Sheet ID	Ice Sheet Thickness (mm)	Pixel Area	Pixel Volume	Pixels per mm	Volume (mm^3)	Analysis Conducted
1	3	1D	1.5	20225	6008	96	0.66	LCMS, qPCR
2	6	1B	2	19098	14797	103	1.40	LCMS, qPCR
3	7	1B	2	3265	2579	105	0.23	LCMS, qPCR
4	12	2B	2	43362	40788	51	15.46	LCMS, qPCR, HTS
5	14	2B	2	9778	13537	140	0.69	LCMS, qPCR
6	15	2B	2	5758	3801	140	0.19	LCMS, qPCR
7	23	4A	3	36921	42670	40	26.76	LCMS, qPCR
8	25	5E	3	27883	30095	61	8.13	LCMS, qPCR, HTS
9	27	5H	3.5	72181	116723	63	29.43	LCMS, qPCR, HTS
10	29	5H	3.5	56869	52628	85	7.28	LCMS, qPCR
11	30	5G	3.5	13707	10973	61	2.97	LCMS, qPCR, HTS
12	31	5G	3.5	40847	43036	54	14.92	HTS
13	32	4B	3.5	15514	20666	56	6.58	LCMS, qPCR, HTS
14	33	2D	2.5	60212	56489	38	39.93	LCMS, qPCR, HTS
15	34	2D	2.5	34732	30711	89	3.90	HTS
16	37	2C	2.5	26685	19192	84	2.72	HTS
17	49	5D	3	35915	53138	40	33.79	LCMS, qPCR, HTS
18	50	5D	3	26036	23349	37	16.70	LCMS, qPCR, HTS
19	51	5D	3	41374	60700	93	6.97	HTS
20	52	5D	3	29528	34669	75	6.20	HTS
21	53	5D	3	19141	32120	135	1.75	HTS
22	55	5D	3	9215	7507	135	0.41	LCMS, qPCR
23	56	5D	3	24775	24115	110	2.01	LCMS, qPCR
24	58	5F	3	17122	13718	58	4.03	LCMS, qPCR, HTS
25	59	5F	3	12437	13243	107	1.15	LCMS, qPCR, HTS

26	61	2E	3	13704	12296	65	2.88	LCMS, qPCR, HTS
27	62	2E	3	9036	4956	82	0.74	LCMS, qPCR, HTS
28	65	3C	2.5	7406	8672	82	1.30	LCMS, qPCR
29	66	3C	2.5	19757	26466	89	3.37	HTS
30	70	3C	2.5	15295	14989	79	2.38	HTS
31	72	3C	2.5	7844	6548	82	0.98	LCMS, qPCR
32	73	3C	2.5	9307	6000	96	0.66	LCMS, qPCR

LCMS = liquid chromatography mass spectrometry analysis for microcystin concentration; qPCR = quantitative polymerase chain analysis for concentration of microcystin-producing *Microcystis*; HTS = high-throughput sequencing of the cyanobacterial internal transcribed spacer gene to assess *Microcystis* population diversity.

Table 2. Microcystin quota information for relevant colony samples.

Colony ID	Ice Sheet ID [a]	Colony Volume (mm^3)	MC Concentration (ng/mL)	*mcyE* Concentration (Copies/mL)	MC Quota (fg/Toxic Cell)
3	1D	0.66	1,943	833,724,153	2.3
6	1B	1.40	3,378	348,986,248	9.7
7	1B	0.23	201	3,270,700	61.4
12	2B	15.46	13,802	1,767,185,139	7.8
14	2B	0.69	1,358	129,226,089	10.5
15	2B	0.19	572	43,086,887	13.3
23	4A	26.76	36,946	189,632,051	194.8
25	5E	8.13	5,764	342,795,027	16.8
27	5H	29.43	28,615	917,401,710	31.2
29	5H	7.28	8,509	903,981,200	9.4
30	5G	2.97	5,970	937,765,820	6.4
32	4B	6.58	8,428	386,074,613	21.8
33	2D	39.93	33,404	389,392,555	85.8
49	5D	33.79	35,691	653,422,733	54.6
50	5D	16.70	24,015	225,007,350	106.7
55	5D	0.41	1,295	135,094,427	9.6
56	5D	2.01	3,710	51,860,037	71.5
58	5F	4.03	16,211	544,497,945	29.8
59	5F	1.15	4,510	128,839,838	35.0
61	2E	2.88	4,861	239,371,820	20.3
62	2E	0.74	4,359	164,464,003	26.5
65	3C	1.30	2,550	430,531,930	5.9
72	3C	0.98	2,444	152,240,662	16.1
73	3C	0.66	1,685	279,687,773	6.0

[a] The ice sheet number corresponds to the collection site/time (see Supplementary Table S4 for more information). MC = microcystin; *mcyE* = microcystin synthase gene E.

Table S3: Sample information for high-throughput sequencing of the cyanobacterial internal transcribed spacer (ITS) gene for relevant colony samples.

Colony ID	Ice Sheet ID[a]	Colony Volume (mm^3)	Number of Reads for *Microcystis* ITS Operational Taxonomic Units (OTUs)										
			OTU_8	OTU_1	OTU_2	OTU_3	OTU_7	OTU_6	OTU_13	OTU_4	OTU_436	OTU_5	Total
12	2B	15.46	348	3	1	0	0	0	0	0	0	0	352
25	5E	8.13	1,496	53	1	1	2	0	0	0	0	0	1,553
27	5H	29.43	1,153	55	9	0	0	2	0	0	0	0	1,219
30	5G	2.97	444	153	19	1	0	0	0	2	0	0	619
31	5G	14.92	1,169	52	2	1	0	0	3	0	0	0	1,227
32	4B	6.58	1,195	48	2	0	0	0	0	0	2	0	1,247
33	2D	39.93	1,248	0	0	1	0	0	0	0	0	0	1,249
34	2D	3.90	1,230	0	0	0	0	1	0	0	0	0	1,231
37	2C	2.72	502	2	0	0	0	0	0	0	0	0	504
49	5D	33.79	613	15	1	1	0	0	0	0	0	0	630
50	5D	16.70	1,111	15	1	0	0	0	2	0	0	0	1,129
51	5D	6.97	341	5	1	0	0	0	0	0	0	0	347
52	5D	6.20	955	1	0	1	0	0	0	0	0	0	957
53	5D	1.75	668	42	2	0	0	0	0	0	0	0	712
58	5F	4.03	815	35	3	0	0	0	0	0	0	0	853
59	05F	1.15	787	86	4	0	0	0	0	0	0	2	879
61	2E	2.88	380	1	0	0	0	0	0	0	0	0	381
62	2E	0.74	367	0	0	0	0	0	0	0	0	0	367
66	3C	3.37	330	2	0	0	0	0	0	0	0	0	332
70	3C	2.38	375	0	0	0	0	0	0	0	0	0	375

[a] The ice sheet number corresponds to the collection site/time (see Supplementary Table S4 for more information).

.

Table S4: Information on sample collection time and location.

Collection [a]	Date	Time	Site Name	Latitude	Longitude
1	12/04/2014	1:02 pm	Pontoon	−41.27166	173.294077
2	13/04/2014	4:05 pm	Boat Bay	−42.40908	173.582840
3	13/04/2014	4:47 pm	Pontoon	−42.40874	173.583094
4	14/04/2014	11:04 am	Launch Bay	−42.40859	173.578876
5	14/04/2014	11:22 am	Launch Bay	−42.40871	173.578716

[a] Collection numbers correspond to the Ice Sheet ID.

Figure S1. Automated pixel selection for colony images where images alternate between the raw image and the selected area.

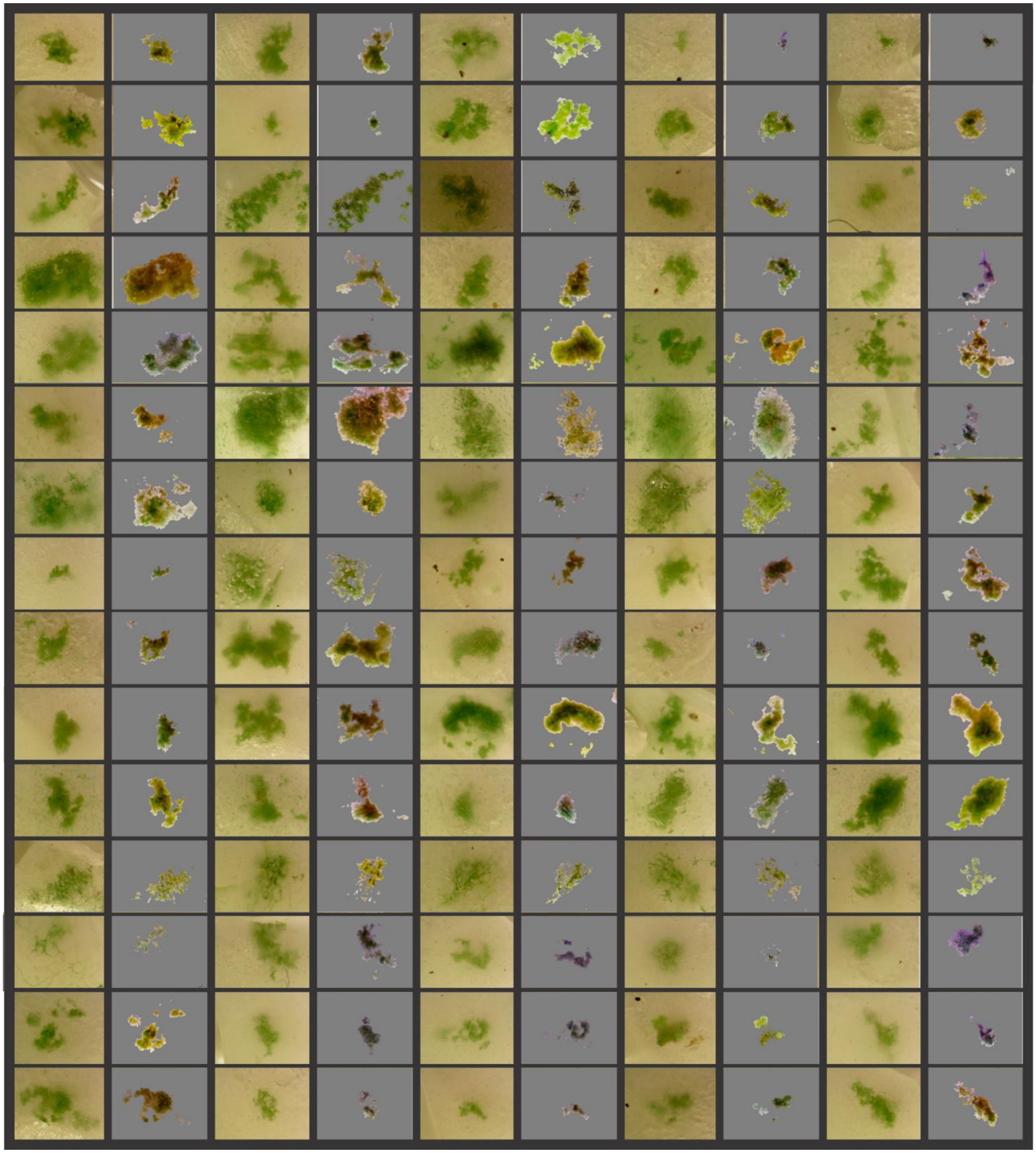

Supplement: Supplementary file 1 [file toxins-11-00435-s001.pdf]
